# Therapeutic Action of Carbolyzed Camphor

**Published:** 1877-03

**Authors:** 


NEW OBSERVATIONS CONCERNING THE THERAPEUTIC ACTION OF CARBOLIZED CAMPHOR.—Dr. Soulez publishes, in *La France Medicale,* of January 11, 1877, a series of cases in which he successfully employed the carbolized camphor. This antiseptic, of which he speaks so highly, is obtained by dissolving nine grammes of the crystals of carbolic acid in one gramme of alcohol; to two grammes of this solution is then added twelve grammes of powdered camphor—the syrup-like liquid which results is the carbolized camphor. For dressings the carbolized camphor is reduced (one part to nineteen of olive oil or oil of sweet almonds), and in the mixture are saturated square pieces of lint, which are then applied, one over the other, to the wound; these are covered by a sheet of thin india-rubber, and on this, again, dry lint is bound with a bandage. M. Soulez claims for this dressing three great advantages: suppression of pain after the operation, absence of febrile reaction, and, finally, rapid cicatrization.—*Clinic.* J. L. A.

